# The importance of side branches of glycosylphosphatidylinositol anchors: a molecular dynamics perspective

**DOI:** 10.1093/glycob/cwac037

**Published:** 2022-10-03

**Authors:** Pallavi Banerjee, Daniel Varon Silva, Reinhard Lipowsky, Mark Santer

**Affiliations:** Department of Theory and Biosystems, Max Planck Institute of Colloids and Interfaces, Potsdam 14476, Germany; Mathematisch-Naturwissenschaftlichen Fakultät, University of Potsdam, Potsdam 14476, Germany; Department of Theory and Biosystems, Max Planck Institute of Colloids and Interfaces, Potsdam 14476, Germany; Department of Theory and Biosystems, Max Planck Institute of Colloids and Interfaces, Potsdam 14476, Germany; Mathematisch-Naturwissenschaftlichen Fakultät, University of Potsdam, Potsdam 14476, Germany; Department of Theory and Biosystems, Max Planck Institute of Colloids and Interfaces, Potsdam 14476, Germany

**Keywords:** conformation, GFP, glycan recognition, GPI, molecular dynamics

## Abstract

Many proteins are anchored to the cell surface of eukaryotes using a unique family of glycolipids called glycosylphosphatidylinositol (GPI) anchors. These glycolipids also exist without a covalently bound protein, in particular on the cell surfaces of protozoan parasites where they are densely populated. GPIs and GPI-anchored proteins participate in multiple cellular processes such as signal transduction, cell adhesion, protein trafficking and pathogenesis of Malaria, Toxoplasmosis, Trypanosomiasis and prion diseases, among others. All GPIs share a common conserved glycan core modified in a cell-dependent manner with additional side glycans or phosphoethanolamine residues. Here, we use atomistic molecular dynamic simulations and perform a systematic study to evaluate the structural properties of GPIs with different side chains inserted in lipid bilayers. Our results show a flop-down orientation of GPIs with respect to the membrane surface and the presentation of the side chain residues to the solvent. This finding agrees well with experiments showing the role of the side residues as active epitopes for recognition of GPIs by macrophages and induction of GPI-glycan-specific immune responses. Protein-GPI interactions were investigated by attaching parasitic GPIs to Green Fluorescent Protein. GPIs are observed to recline on the membrane surface and pull down the attached protein close to the membrane facilitating mutual contacts between protein, GPI and the lipid bilayer. This model is efficient in evaluating the interaction of GPIs and GPI-anchored proteins with membranes and can be extended to study other parasitic GPIs and proteins and develop GPI-based immunoprophylaxis to treat infectious diseases.

## Introduction

Glycosylphosphatidylinositols (GPIs) are complex glycolipids ubiquitous in eukaryotes that are bound to the outer leaflet of the cell membrane (Ferguson et al. 1985; Homans et al. 1988; Ferguson 1999). GPIs are added in the endoplasmic reticulum as a post-translational modification to the C-terminus of proteins having the corresponding peptide signal. The structure of a GPI is characterized by the unique conserved pseudopentasaccharide core: Man*−*α(1 *→* 2) *−* Man*−*α(1 *→* 6) *−* Man*−*α(1 *→* 4) *−* GlcN* −*α(1 *→* 6) *− myo*-inositol, a phosphoethanolamine bridging the anchored protein and the glycan, and a lipid tail that inserts into the cell membrane. This linear core is modified in a cell- and tissue-dependent manner by additional sugars or phosphoethanolamine units. The complexity of the GPI core together with the modifications on the glycan and lipid moieties and the diversity of anchored proteins suggest a variety of functions for these glycolipids. Apart from their function as a stable anchor for proteins, GPIs and GPI-anchored proteins (GPI-APs) have been associated with key cellular processes such as signal transduction, cellular adhesion and communication, protein sorting and trafficking (Low and Saltiel 1988; Tachado et al. 1997; Karagogeos 2003; Mayor and Riezman 2004). This multifunctionality of the GPI anchor makes it a subject of great interest.

Although GPI glycolipids are known for more than 30 years (Ferguson et al. 1985; Homans et al. 1988; Low and Saltiel 1988; Homans et al. 1989; Ferguson 1999), yet there are still ongoing controversial debates about the GPI’s conformation and orientation with respect to cell membranes, its role in raft formation and protein sorting, as well as its antigenic activity. For example, some studies suggest that GPIs stand upright avoiding direct contact with the membrane (Paulick et al. 2007a, 2007b), whereas other studies indicate that GPIs lie in close proximity to the membrane pulling down the attached proteins (Homans et al. 1989; Rademacher et al. 1991; Lehto and Sharom 2002). Preferential partitioning of GPI-anchored proteins into liquid-ordered domains, or rafts, of cell membranes is also unclear (Schuck and Simons 2006; Eggeling et al. 2009; Sevcsik et al. 2015). Such controversial reports arise mainly because of the heterogeneous composition of the glycan part even with the same protein attached (microheterogeneity), which hampers functional and physico-chemical characterization. Thus, there is only a limited number of experimental studies of GPIs. The chemical synthesis of GPIs and GPI-anchored proteins has emerged as an alternative to molecules isolated from in vivo samples (Roller et al. 2020), but this synthetic approach is rather challenging, especially to obtain GPIs consisting of unsaturated lipids and natural GPI-anchored proteins (Tsai et al. 2012; Kinoshita and Fujita 2016).

GPIs participate actively in the modulation of the host immune system during infections by protozoa such as in toxoplasmosis, malaria, and trypanosomiasis (Taylor and Hooper 2011). The membrane of the parasites causing these diseases is characterized by the presence of multiple copies of GPI-anchored proteins and free GPIs that together serve as a protective coat or as immunomodulators. Experimental studies conducted both in vitro and in vivo have shown the activity of GPI modifications to trigger specific immune responses. Debierre-Grockiego and coworkers demonstrated the binding of human-galectin-3 to *Toxoplasma gondii* GPIs using surface plasmon resonance (SPR) and showed a galectin-3 dependent recognition of parasitic GPI glycans by macrophages to induce TNF-α production (Debierre-Grockiego et al. 2010). A synthetic GPI from *T. gondii* has been used as a diagnostic biomarker to differentiate between latent and acute stages of Toxoplasmosis (Gotze et al. 2014). Similarly, immunization with galactose-containing GPI glycan induced TNF-α production in mice that abrogated the pathology and prolonged animal survival to an infection with trypanosomes (Magez et al. 2002). Immunization with synthetic GPI has also shown potential to induce protection against an infection by *Plasmodium falciparum* and has been suggested as a candidate for vaccine development against malaria (Schofield et al. 2002).

Although the biological relevance of GPI anchors for cells is well known, the challenges associated with their isolation or synthesis have so far precluded establishing a general structure–function relationship. Molecular dynamics (MD) serves as a useful complementary technique to unravel structural and dynamical aspects of a system with atomic resolution. Some examples of modeling GPI-APs in membrane-like environments include analyses within the nanosecond timescale of GPIs, such as that of the Variant Surface Glycoprotein of *Trypanosoma brucei* (Homans et al. 1988), the NETNES glycopeptide of *Trypanosoma cruzi* (Chiodi and Verli 2013), and the human prion protein (Zuegg and Gready 2000; DeMarco and Daggett 2009). However, considering the conformational flexibility of GPIs, longer simulation times, at least up to the microsecond timescale, are required for adequate sampling. Using microsecond-long MD simulations, Wu et al. reported a stabilization of the secondary structure of the prion protein through mutual interactions between the protein, GPI anchor, N-glycans and the membrane (Wu et al. 2015). Li et al. (2018) conducted biased MD simulations to report spontaneous insertion of GPIs into the liquid-ordered phase of the membrane. These studies provided information about the interplay between GPI and membranes, but there is no clear understanding of the role of GPI modifications and their effect on the attached protein.

**Fig. 1:**
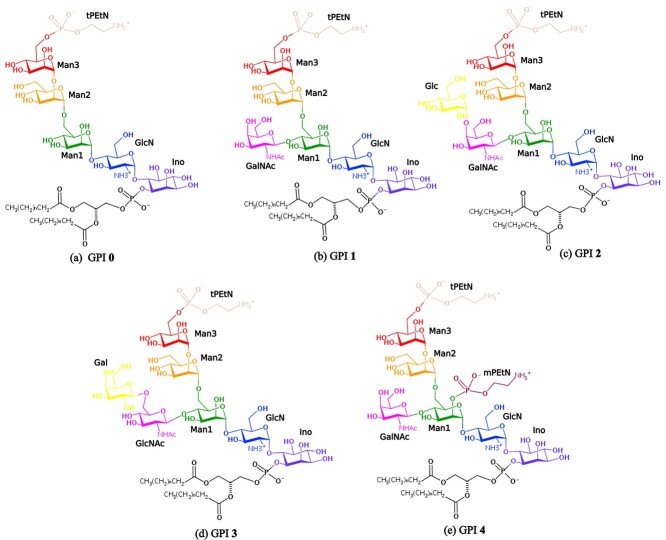
Chemical structures of 5 GPIs labeled 0–4 in this study: (a) GPI 0 corresponding to the GPI core (b) GPI 1 of *T. gondii*, (c) GPI 2 representing the LMW antigen of *T. gondii*, (d) GPI 3 of *T. congolense*; and (e) GPI 4 of human eCD59 protein. The conserved segment, the GPI core, (GPI 0), contains residues: Ino, GlcN, and 3 mannoses; Man1, Man2 and Man3. The terminal PEtN at Man3 is denoted by tPEtN. The side branches of the branched GPIs are as follows: GalNac in GPI 1, GalNac and Glc in GPI 2, GlcNac and Gal in GPI 3, GalNac and the middle PEtN at Man1 called mPEtN in GPI 4.

In our previous work, we analyzed the conformational behavior of GPI glycan (without the lipid tail) in solution using the GLYCAM06 force-field, and reported that a simulation time of at least 1 μs is required to achieve sufficient sampling (Wehle et al. 2012). In a subsequent study, we anchored the GPI core glycan having a lipid tail to the green fluorescent protein (GFP) and showed that the GPI flops down on the bilayer surface making extensive contacts with the lipid headgroups (Banerjee et al. 2018). In the present investigation, we extend our model to study GPIs from the parasites *T. gondii* (GPIs 1 and 2) and *Trypanosoma congolense* (GPI 3) as well as the GPIs found in mammals (GPI 4), see Figure 1. To connect up to our previous work, we investigate the protein-free GPIs first and then observe the change when attaching GFP. We refer to the protein-attached GPI as GFP–GPI in this paper. In both situations, we systematically study the impact of the various side chain combinations displayed in Figure 1 on the inclination and orientation of the GPI backbone and the extent of hydration of the glycan residues in both DMPC and POPC bilayer patches. In turn, we characterize the interaction of the glycan residues with the attached protein. Overall, our results explain the accessibility of some glycan epitopes and the specific immune responses triggered by parasitic GPIs, elucidating the difference in the topology of parasitic and human GPIs. They further corroborate that the orientation and accessibility of the attached protein is sensitive to both the GPI modifications and the bilayer composition.

## Results

### Conformation and topology of protein-free GPIs

**Fig. 2:**
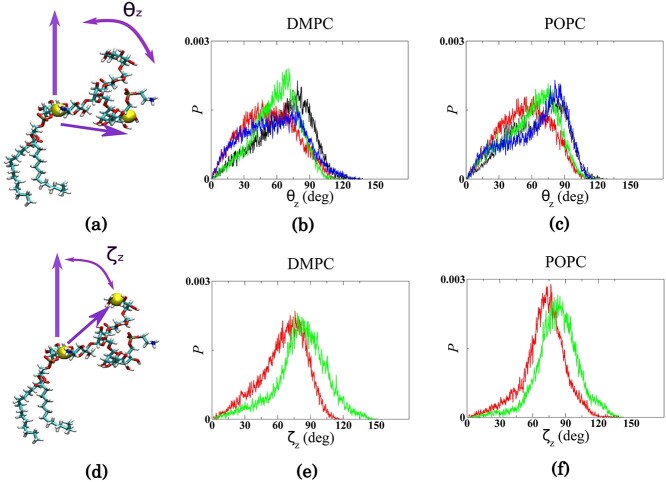
Comparison of tilt angles between all 4 GPI variants inserted in (b,e) DMPC and (c,f) POPC bilayers. The tilt angle *θ_z_* of the GPI core is defined in (a) and that of the alternative tilt angle *ζ_z_* comprising the side branch is defined in (d). The yellow beads depict the end points of the position vector. In (b) and (c), the distributions of the angle *θ_z_* are plotted for GPI 1 (black), 2 (red), 3 (green), and 4 (blue). In (e) and (f), the distributions of the angle *ζ_z_* are plotted for GPI 2 (red) and 3 (green). *P* on the *y*-axis represent probability density.

We construct the GPI model by combining the AMBER force-fields—GLYCAM06 (Kirschner et al. 2008) for the GPI glycan and Lipid14 (Dickson et al. 2014) for the lipid tail. To address the case of the free GPIs, 3 1 μs long MD simulations each of the branched GPIs 1–4, see Figure 1, inserted in (8 × 8) DMPC and POPC bilayer patches showed a flopped-down orientation for all GPI structures. To determine this orientation, we calculated the tilt angles *θ_z_* for each GPI with respect to the bilayer using the position vector connecting the end points of the GPI core, pointing from atom C6 of Ino to atom C4 of Man3, and the bilayer normal (*z*-axis), see Figure 2(a). For all GPIs with a glycan branch, we also calculated an alternative tilt angle denoted by *ζ_z_* using the atom C6 of Ino and atom C4 of Glc/Gal attached to Man1 residue as end points for the position vector, see Figure 2(d). The distributions of the tilt angle *θ_z_* for the GPIs are quite broad, reflecting their structural flexibility, however, they largely peak around 70–80° as is seen more prominently in GPIs 1 (black) and 3 (green), see Figure 2(b) and (c). The distributions for the alternative tilt angle *ζ_z_* of GPI 2 and GPI 3 are narrower, with pronounced peaks around 70–80° and the peak of GPI 3 distribution being shifted towards higher values, see Figure 2(e) and (f). These results demonstrate that branched GPIs largely prefer to flop down on the membrane bringing either the GPI core or the side branch into contact with it. Additionally, we assessed the effect of the water model on the GPI conformation to observe drastic differences if any. To this end, we simulated GPI 0 in TIP5P water to compare with TIP3P water. Figure S1 in the SI shows that irrespective of the water model, GPI is still flopped down on the bilayer.

**Fig. 3:**
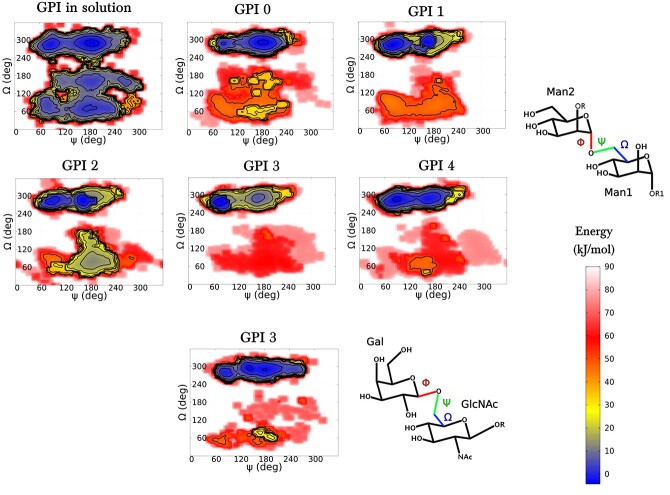
Two-dimensional free-energy landscapes as functions of dihedral angles ψ and Ω for the (1 *→* 6) linkages in the protein-free GPI variants embedded in DMPC bilayer. Man2-α(1 *→* 6)-Man1 dihedral plots are shown for all GPIs—0, 1, 2, 3, and 4—in comparison to GPI glycan in solution, and an additional gal-β(1 *→* 6)-GlcNAc plot is shown for GPI 3. In the chemical structures beside the plots, the bond colors correspond to the colored symbols for dihedrals φ, ψ and Ω, the definitions of which can be found in the paragraph before equation (1).

The overall 3D conformation of a glycan is largely governed by the glycosidic linkages. These linkages can adopt different values depending on the type of monosaccharide, the anomer type, and also the type of linkage (1 *→* 2*,* 1 *→* 3*,* 1 *→* 4*,* 1 *→* 6). A glycosidic linkage connecting 2 sugar residues is usually represented with 2 torsion angles—*φ*(*C*_2_*, C*_1_*, O_x_^′^ , C_x_^′^* ) and *ψ*(*C*_1_*, O_x_^′^ , C_x_^′^ , C_y_^′^* )—and an additional angle Ω(*O*_6_*^′^ , C*_6_*^′^, C*_5_*^′^ , O*_5_*^′^* ) (relevant for (1 *→* 6) linkages), see Figure 3 for their pictorial representation. Ramachandran-like (*φ, ψ*) and (*ψ,* Ω) free-energy plots were generated for all the glycosidic linkages of protein-free GPI variants studied in DMPC and POPC bilayers. The free energy associated with a probability distribution for the dihedral pair (*φ, ψ*) is obtained via:

(1) \begin{equation*} F\left(\varphi, \psi \right)=-{k}_B TlnP\left(\varphi, \psi \right) \end{equation*}

where *k_B_* is the Boltzmann constant, *T* is temperature, and *P* (φ*,* ψ) is the probability distribution for a dihedral angle pair (φ*,* ψ). We observed that in comparison with the GPI conserved core, the distributions of all the corresponding torsions across all the GPI variants are quite similar, except for the α(1 *→* 6) linkage connecting the Man2–Man1 units. This (1 *→* 6) linkage is more flexible due to the presence of a primary hydroxyl group in the glycosidic bond and the additional rotation at the methylene group. Moreover, the Gal-β(1 *→* 6)-GlcNAc linkage that is the side branch component of GPI 3 also shows considerable flexibility. The variation in the Man2-α(1 *→* 6)-Man1 linkage across all GPI variants and the flexibility of the Gal- β(1 *→* 6)-GlcNAc linkage in GPI 3 are together depicted in the Ramachandran-like (ψ*,* Ω) plots in Figure 3. In a previous study of the GPI tetrasaccharide core in solution, we demonstrated the flexibility of the α(1 *→* 6) linkage connecting the residues Man1 and Man2, serving as a hinge between the 2 relatively rigid disaccharides on either side (Man3–Man2 and Man1–GlcN) (Wehle et al. 2012). Out of the possible staggered rotamer states, *gg* (240° *<* Ω *<* 360°) was seen to be the most populated, with only minor traces of *gt* (0° *<* Ω *<* 120°) and *tg* (120° *<* Ω *<* 240°), as a consequence of the gauche effect (Kirschner and Woods 2001). Upon comparing GPI core in solution with GPI 0 in the bilayer in Figure 3, one can say that anchoring reduces the flexibility of the (1 *→* 6) linkage, as the free energy seems to have shifted more towards the *gg* rotamer in the anchored GPI.

The rotamer populations for Man2–Man1 of all GPIs are listed in Table 1. For GPIs 0, 1, and 4, the population ratios are consistent with *gg* being the highest, followed by *gt* and negligible traces of *tg*. As we move towards GPIs with an extra side branch residue, GPIs 2 and 3, the population of the *gt* rotamer relatively goes up, even to the same level as that of *gg* for GPI 2 in POPC and more than *gg* for GPI 3 in POPC. These results suggest that the lipid bilayer type certainly has some notable impact on the conformation of the GPI anchor, particularly at the flexible linkages. The *β*(1 *→* 6) Gal-GlcNAc linkage presents a profile different from the Man2-Man1 linkage in both the *ψ* torsion and rotamer populations. The G al-GlcNAc rotamer population distribution (*gg/gt/tg*) in DMPC is (56/36/9)% and in POPC is (78/20/2)%, again showing highest preference for *gg*. Both *ψ* and Ω appear more flexible in Gal-GlcNAc than in the Man2–Man1 linkage, consistent with Gal as a terminal residue. Still, rotamer populations are dominated by the *gauche* conformers over *anti* in both Man2–Man1 and Gal-GlcNAc linkages, consistent with the general trend observed in manno- and gluco-pyranosides (Patel et al. 2014). The free-energy profiles for the rest of the glycosidic linkages of all the GPIs can be found in Figure S2 of the SI.The terminal phosphoethanolamine (tPEtN) that is attached to Man3 (see Fig. 1) is found on all GPI variants, whether with or without protein. It serves to attach proteins to the GPI through an amide bond formation between the C terminal end of the protein and the amine group of tPEtN. As the tPEtN group is also present for free GPI, we show here for completeness a comparative analysis of how the presence of the tPEtN linker affects the conformation of the GPI. We therefore simulated protein-free GPIs – GPIs 0, 1, 2, and 3 with the terminal PEtN group removed in both DMPC and POPC bilayers. These systems were also subjected to 3 parallel 1 μs long runs, as the previous systems with the linker attached. The comparison is described in detail in Section S1.3 of the SI. We observed that the conformation, by and large, remains the same as the tPEtN-attached GPIs, except for the terminal Man3–Man2 linkage.

**Table 1:** Rotamer populations of the α*1 6* linked Man2–Man1 linkage across all the protein-free GPI variants inserted separately in DMPC and POPC bilayers.

GPI	DMPC			POPC		
	gg%	gt%	tg%	gg%	gt%	tg%
0	66	25	9	75	22	3
1	76	23	1	91	7	2
2	59	33	8	41	41	18
3	83	13	4	37	54	9
4	78	19	3	92	8	0

### Significance of GPI side branches

**Fig. 4:**
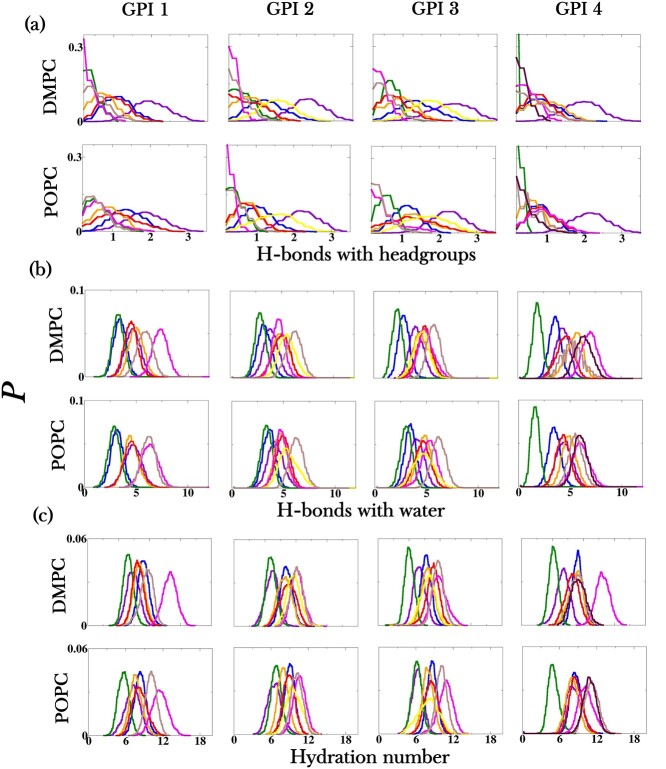
Probability distributions of the (a) number of H-bonds formed between each residue of the GPI variants and the lipid headgroup region, (b) number of H-bonds between each GPI residue and water, and (c) hydration number of each GPI residue. The plots show data for GPI variants 1, 2, 3, 4 inserted in DMPC and POPC bilayers. *P* on the *y*-axis of all the plots represents the probability density. The data in the plots are normalized. Refer Figure 1 for the color coding of the residues in respective GPIs.

The extent of accessibility of the glycan residues can be estimated by their degree of interaction with the lipid heads of the bilayer and with water. These glycan interactions are determined in 2 ways: (i) by recording the number of hydrogen bridges formed by each GPI residue with the lipid headgroup and with water, (ii) by counting the hydration number of each GPI residue. The presence of several hydroxyl groups in carbohydrates makes them highly prone to hydrogen bonding (H-bonding). Figure 4(a) shows that in all parasitic GPIs 1, 2 and 3, GalNAc/GlcNAc, Man1 and tPEtN hardly form H-bonds with the polar headgroups of lipids. In human GPI 4, similar behavior was observed except that Man1 and mPEtN interact significantly less with the bilayer than does GalNAc. The degree of interaction of GPI residues with water is indicated in Figure 4(b) and (c). Figure 4(b) shows the probability distributions of H-bonds formed by each GPI residue with water molecules, and Figure 4(c) contains the hydration number of each residue for every GPI variant, where hydration number means the number of water oxygens lying within a radius of 0.3 nm from every atom of the residue being considered. GPI side branch residues directly attached to Man1, that is either GalNAc or GlcNAc or mPEtN, are seen to relatively orient more into the solvent subphase compared to the rest of the residues. Although GalNAc/GlcNac bear notable H-bonding potential, they scarcely form H-bonds with the bilayer, see Figure 4(a). However, Figure 4(b) and (c) shows that in all cases they are highly interactive with water through H-bond formations, more compared to the other sugar residues. The same can be inferred from the distance distribution plots in Figure S4, where GalNAc/GlcNAc lie slightly more outward, and therefore more exposed, compared to the other residues. Even the terminal side branch residue Glc in GPI 2 and the PEtN linkers in all GPIs are comparatively more hydrated than the remaining residues. In GPI 4 that is found in mammals/humans, although both the PEtN residues appear as almost equally hydrated, see Figure 4(c), the H-bonding profiles in Figure 4(b) bring out the difference between the 2 in that mPEtN forms more H-bonds with water than does tPEtN. Besides, in the distance distribution profiles of Figure S4, mPEtN is more outward or more solvent-facing than tPEtN. These findings suggest that, through comparatively more exposure to the solvent, the side chain residues, whether saccharide units or PEtN linkers, branching out from Man1 may participate in GPI recognition.

The terminal side branch residues of GPIs 2 and 3 are placed differently w.r.t. the lipid bilayer, despite the fact that both the GPIs are of the same length and size. Gal of GPI 3 is less exposed to water than Glc of GPI 2, as can be seen in Figure 4(b) and (c). Figure S4 shows that the former lies more embedded in the lipid headgroup region than the latter. A plausible reason behind this difference could be the longer and flexible *β*(1 *→* 6) linkage in the side branch composition of GPI 3, which allows it the conformational freedom to interact with the lipid heads, compared to its stiffer *α*(1 *→* 4) counterpart in GPI 2 (compare Figure 1(c) and (d)). The difference in flexibility of the side-branch glycosidic linkages, that is between Glc-GalNAc of GPI 2 and Gal-GlcNAc of GPI 3, is illustrated in the free-energy plots of Figure S2. Thus, seemingly minute differences in structure, such as in linkage types, can affect the solvent-accessibility of GPI glycans. Additionally, it is also apparent that the type of membrane makes a difference to the accessibility of the residues. All GPIs interact with the lipid headgroup region more in POPC than in DMPC as is seen in Figure 4(a). Moreover, Figure 4(b) and (c) shows that GPI residues are less solvent-interactive when inserted in POPC than in DMPC. This discrepancy arises from the different areas-per-lipid of DMPC and POPC bilayers, resulting in less packing of lipids in POPC than in DMPC. Such discrepancy was previously observed with the GPI core simulations in our previous work as well (Banerjee et al. 2018).

### GPI-anchored GFP

In our previous work, we had seen that anchorage to the lipid bilayer affects the conformational flexibility of the GPI glycan (Banerjee et al. 2018). The central *α*(1 *→* 6) linkage, for example, is forced to assume primarily its *gg* rotamer. Additionally, it was demonstrated that with the presence of the flexible phosphoethanolamine linker (tPEtN), the attached protein GFP retained considerable re-orientation flexibility, which is, in principle, sufficient to establish protein-glycan contacts. With the variety of GPI variants modeled in this work it is now possible to look at protein–glycan interactions in more detail. These interactions are expected to be important for the structural stability of some attached proteins (Ikezawa 2002; Ferguson et al. 2017). We study the protein-glycan interactions in 2 ways: first, we analyze how the conformational flexibility of the GPI glycan itself changes when a protein is attached as compared to the case of free GPIs; in addition, the statistics of protein–glycan contacts are worked out to assess the potential for impacting protein stability. To understand the nature/extent of interactions of an attached protein with GPI, we chose the GFP, which is not a naturally occurring GPI-anchored protein, however, due to its easy availability and fluorescent nature, it has been extensively used for experimental studies of GPIs (Prior et al. 2003; Sharma et al. 2004; Sevcsik et al. 2015). The structure file of GFP was taken from its crystal structure, RCSB id – 1EMA (Ormo et al. 1996), consisting of 226 amino acid residues. We designed the protein GFP with the AMBER-ff14SB force-field (Maier et al. 2015). The protein was attached to the GPI at the terminal mannose residue Man3 through a phosphoethanolamine (tPEtN) linker, at the amine group of the molecule, see Figure 1.

#### Impact of the anchored GFP on GPI conformations

**Fig. 5:**
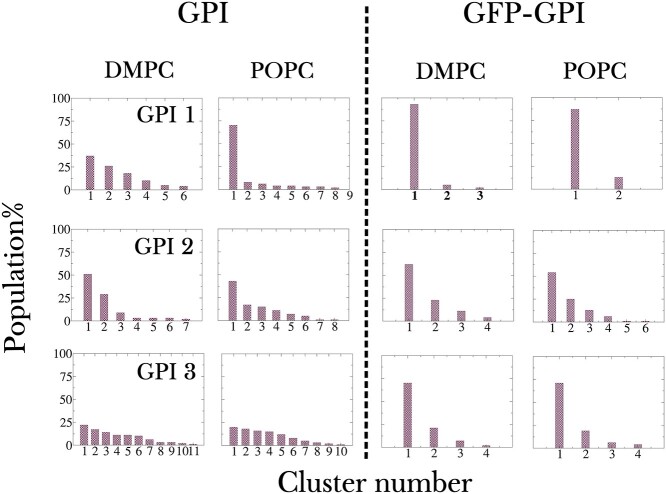
Cluster size distributions for GFP-free and GFP-bound GPIs 1, 2, 3 in both DMPC and POPC bilayers. Note that for the free GPIs both the top and bottom leaflet have been included in the calculation. Cluster number refers to the denomination of clusters in decreasing order of their population such that cluster 1 is the most populated cluster.

**Fig. 6:**
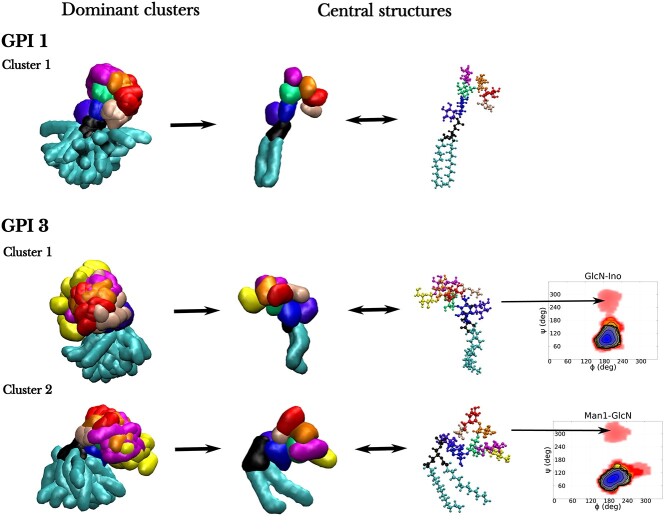
Conformers of the dominant clusters of GPIs 1 and 3 inserted in DMPC bilayer. Around 20 members of cluster 1 for GPI 1 and clusters 1 and 2 for GPI 3 obtained from clustering analysis on 6 μs worth simulation data are superimposed w.r.t. the residues GlcN-Ino-PGL on the left. On their right are shown the central structures of the corresponding clusters in 2 representations, quicksurf and licorice. Color coding for residues follows that of Figure 1. Additionally, for GPI 3 free-energy contour plots of the glycosidic linkages GlcN-Ino and Man1-GlcN are displayed to indicate the regions occupied by the dominant clusters.

Firstly, to assess the effect of the attached GFP on the GPI anchor, we performed cluster analysis on both free GPIs and GFP–GPIs. Cluster analysis filters out conformations that predominantly occur in the simulation trajectories. Each of the concatenated 3μs long MD trajectories of the free GPIs, including those from both the top and bottom leaflet of the bilayer was subjected to the analysis. Considering the high flexibility of GPIs, only the clusters with more than 40 members were analyzed, where members are the structures lying within a cluster, see Section: ‘Cluster size calculation’ in ‘Computational methods’. The population distributions of clusters for each free GPI system, shown in Figure 5, indicate their large conformational flexibility. Out of these, GPI 3 shows extraordinary structural variability, as is evident from the broad distribution in its cluster sizes, where cluster size refers to the number of members in a cluster. This is because of the presence of 2 highly flexible and dynamic (1 *→* 6) linkages, one *α* (main chain) and the other *β* (side chain), in its structure, see Figure 1(d). For a visual representation, the dominant clusters of 2 GPIs, GPI 1 (less flexible) and 3 (more flexible), embedded in DMPC bilayer are displayed in Figure 6. Due to the large spread in its cluster distribution, 2 most dominant clusters 1 and 2 are shown for GPI 3. The cluster conformations have been superimposed on each other by aligning them with respect to the relatively rigid residues GlcN-Ino-PGL. The central structure of the clusters are also displayed in Figure 6. The large volumes spanned by the GPI residues in the clusters are further indicative of their structural flexibility. Besides, the flop-down conformation is confirmed by the conformation of the central structures. Upon measuring the glycosidic torsions of the clusters, it was revealed that the most populated cluster, cluster 1, of the GPIs, except for GPI 3, bore torsion values from all the lowest free-energy wells together, i.e. the overall GPI conformation corresponded with the well depths of the glycosidic torsions, see Figure S2. In the case of GPI 3, although substantial conformational differences were expected at the flexible linkages Man2–Man1 and Gal-GlcNAc, even the relatively rigid linkages showed considerable variation. For instance, the central structure of cluster 1 of GPI 3 in DMPC carried torsional values (*φ, ψ*) = (192,281) for GlcN-Ino and that of cluster 2 carried (*φ, ψ*) = (209,300) for Man1-GlcN, both from seemingly minor energy wells, as illustrated in Figure 6. This shows that in more flexible GPIs, the dominant conformations may occupy less stable values for certain glycosidic linkages.

The same cluster analysis was carried out on GFP–GPIs in both DMPC and POPC bilayers using concatenated 4 μs long MD trajectories with more than 30 cluster members considered for analysis, see Figure 5. Comparison between GPI and GFP–GPI makes it clear that the conformation of GPI is more stable, i.e. it is less flexible, with an attached protein. This difference is most prominent in GPI 3, where with an attached protein not only did the number of clusters reduce but also the size of the largest cluster, cluster 1, significantly rose. All in all, upon attaching GFP, cluster size distributions narrowed down for all the GPI variants. This restricted conformational flexibility of GFP–GPIs is also apparent in the Ramachandran-like plots for the glycosidic torsions shown in Figure S5 of the SI, where the energy wells are less scattered than in free GPIs. Dominant conformers from the most populated clusters of each GPI can be found in Figure S6 of the SI. Protein attachment was seen to inflict maximum conformational deviation with respect to free GPIs at the flexible Man2-*α*(1 *→* 6)-Man1 linkage. For example, the rotamer population was the highest for *gg* in free GPI 0 both in DMPC and POPC bilayer, whereas with attached GFP, the population shifted towards *gt* (compare Figures S2 and S5). There was considerable deviation observed at *ψ* of Man2–Man1 too, going from 2 prominent energy wells in free GPIs to essentially one in GFP–GPIs. The cluster conformations in Figure S6 show that irrespective of the orientation of the attached GFP, whether erect or reclining, the GPI lies close to the membrane with the glycan head spanning across the membrane surface, although the internal arrangement of GPI residues, particularly at the flexible linkages—Man2–Man1 and Gal-GlcNAc–, may vary.

#### Orientation of anchored GFP

**Fig. 7:**
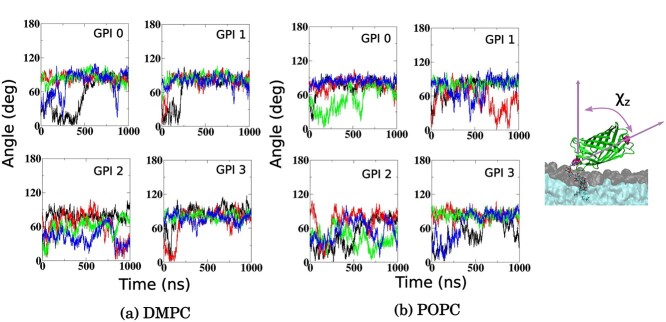
Time evolution of the tilt angle of GFP when attached to 4 different GPIs 0, 1, 2, and 3, as introduced in Figure 1, placed in (a) DMPC and (b) POPC bilayers. For each system, results from 4 independent 1 μs long trajectories (corresponding to 4 different colors) are placed against each other. The cartoon on the right shows the definition of the tilt angle *χ_z_*, which represents the angle between the position vector connecting GFP residues GLN(76) and HIE(135) (shown in pink), and the bilayer normal.

The orientation of GFP with respect to the bilayer was determined by measuring the tilt angle of the axis of the GFP barrel formed with the bilayer normal, see cartoon in Figure 7. The time evolution of the tilt angle in each of the 4 1 μs long trajectories for each GPI variant in DMPC and POPC bilayers is displayed in Figure 7. The tilt angle largely occupies high values *≈*80°, except in GPI 2, where the orientation of GFP was seen to highly fluctuate. The higher degree of fluctuation of tilt angle in GPI 2 compared to GPI 1 shows that the size or molecular weight of GPI affects the orientation as GPI 2 carries 1 additional branched sugar residue. Interestingly, GPI 3, despite having the same size as 2, behaves very differently, in the sense that the tilt angle is quite stable at about 80–90° in the former as opposed to the instability of the latter. This indicates that the orientation of the attached protein could be affected not just by the size of GPI, but also the chemical composition of the side chain residues, at least within the pool of simulation data acquired here. Moreover, GPIs inserted in POPC bilayer exhibit greater fluctuation in tilt angle than those in DMPC bilayer, suggesting that the local distribution of lipids also influences the protein’s presentation. Note that GPI 2 does not naturally come with a protein, but only exists in the protein-free form on the cell surface of the parasite. Nevertheless, it was attached to the protein in this study to facilitate a systematic investigation that may guide future synthetic, in vitro experiments.

#### Interaction of GFP with GPI

**Fig. 8:**
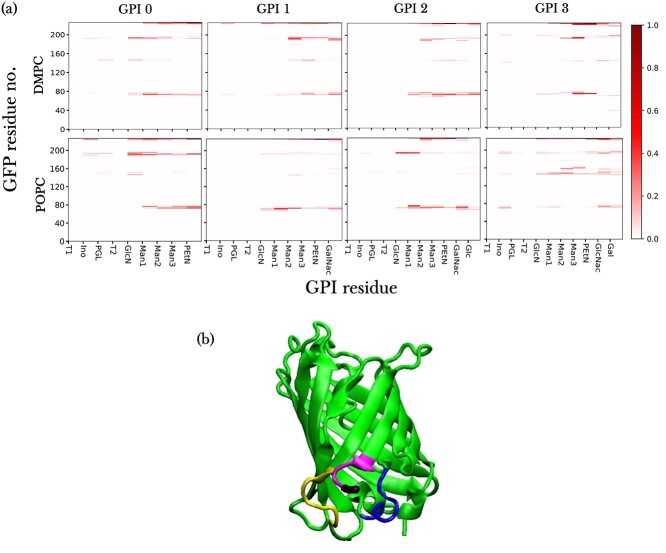
(a) Contact maps of GPI-anchored GFP in DMPC (top row) and POPC bilayers (bottom row) obtained for GPI types 0, 1, 2, and 3. The maps show the relative fraction of frames containing contacts between residues of GFP (*y*-axis) and GPI (*x*-axis). On the *x*-axis, T1 and T2 refer to myristoyl tails of GPI. The scale of contact fractions (color bar) ranges from 0 to 1. Each color box in the map represents the GPI residue to its left and GFP residue under it. (b) GFP residues that are in frequent contact with GPI. Different colors indicate different residue ranges, with residues 69–77 shown in blue, residues 190–196 in yellow, and residues 222–226 in magenta. The C terminal threonine, residue 226, is colored black (at the end of the magenta segment) to indicate the point of attachment to GPI.

**Fig. 9:**
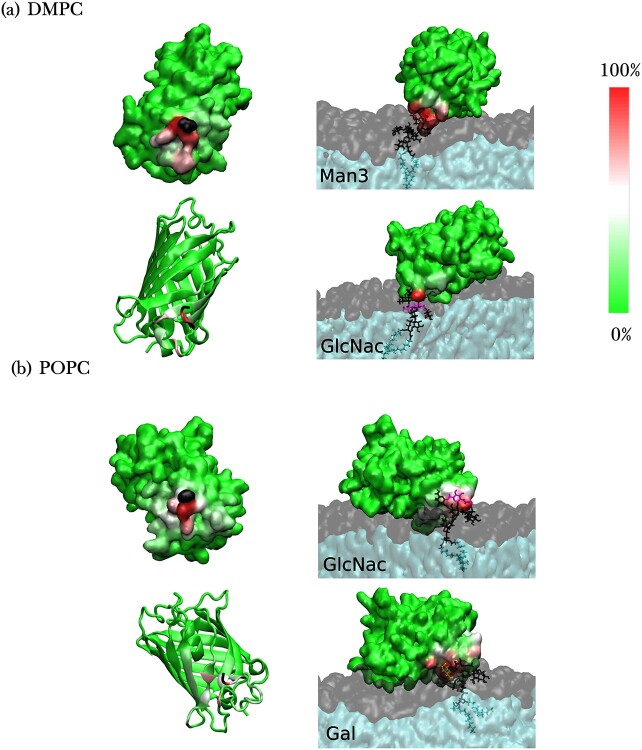
Frequency of contacts between atoms of the whole GPI 3, displayed in panel (d) of Figure 1, and GFP mapped onto the structure of GFP in 2 different representations (quicksurf and ribbon), both for (a) DMPC and (b) POPC bilayers. Here the C-terminal residue, threonine, is colored in black to depict the point of attachment of GPI. In the right column of images, the color-coded contact frequency for the 2 most protein-interactive residues, as mentioned in the lower left corners of the images, of the GPI variants is mapped onto representative conformations of the corresponding GPI- anchored GFP in bilayers. The residues in question are color-coded according to Figure 1 while the rest of the GPI glycan is colored black.

Understanding the interactions between the GPI anchor and its attached protein is imperative to their conformational analysis. Just as GPIs form H-bonds with the lipid headgroups, they also do so with the GFP, see Figure S7. Here, more generally we calculated the number of contacts formed between residues of GFP and GPI by counting the atoms of GFP lying within a cutoff of 0.5 nm from the reference GPI residue, according to the approach by Swenson and Roet (2020). Figure 8(a) shows contact maps for residue-wise contacts formed between GFP and GPI variants—0, 1, 2, and 3—in DMPC and POPC bilayers. There is extensive interaction between GFP and all the GPI types which even goes so far as the innermost GPI glycan residue, inositol (Ino), and sometimes even to the phosphoglycerol (PGL) head. There are substantial contacts made with GFP from Man1 upwards till Man3 including the side branches, with GlcN, Ino, and PGL interacting only seldom (in decreasing order) with the protein. The contact maps show that certain regions of the protein consistently interact with all the GPI types. These regions or residue patches that are frequently in contact with the GPI happen to be the disordered loop residues of the GFP barrel, as illustrated in Figure 8(b). The flexibility of these loops, that interact with the already flexible GPI, reflects on the flexible orientation of GFP, see Figure 7. The GPI residues making frequent contacts with the GFP are shown in Figure S8 of the SI for all the GPI variants. These residues usually include the terminal residue Man3, Man2 and some of the side branches. Contact frequency maps are displayed for the glycan residues of GPI 3 making the most and second-most number of contacts with attached GFP in Figure 9. The side branch GlcNAc forms extensive contacts with GFP in both (a) DMPC and (b) POPC, with Gal also participating in (b). Similar maps for the rest of the GPI types are shown in Figure S9 of the SI. The most protein-interactive GPI residues include at least one of the side branch residues in all the parasitic GPIs. GPI 0 has Man1, the residue that branches out to side chains, making the maximal contacts with GFP in both DMPC and POPC. The hydration number profiles show that the side branches are quite distinctly the most hydrated out of all residues and also have the greatest freedom in communicating with the protein, see Figure S10. These results, like those from the free GPIs, are again suggestive of the importance of the side branches in GPI anchors.

## Discussion

Chemical heterogeneity and conformational flexibility of glycolipids like the GPI anchor pose limitations on the experimental investigation of their high-resolution structure in the environment of membranes. As GPI anchors are found abundantly across protozoan parasites, they are of particular interest for vaccine development and therapeutics, for which study of the molecular basis of recognition mechanism serves as an important supplement. MD has proven to be an indispensable tool in probing interactions at the atomic-level of resolution. In our previous work, we constructed a model of the GPI anchor core (GPI 0) by combining the force-fields GLYCAM06 and Lipid14 for MD simulations (Banerjee et al. 2018). In the present work, we have expanded this model by incorporating additional saccharide and phosphoethanolamine units to study 3 different parasitic GPI variants – *Toxoplasma gondii* (GPI 1), *T. gondii* low molecular weight (LMW) (GPI 2), *T. congolense* (GPI 3)—and Human GPI (GPI 4). We have previously demonstrated the flexibility in the solution structure (Wehle et al. 2012) and the membrane form (Banerjee et al. 2018) of the GPI glycan core, where large torsional fluctuations were observed at the Man2-*α*(1 *→* 6)-Man1 linkage, as has also been reported by Chevalier et al. (2005) through NMR and molecular mechanics. In the same study, we also showed that the GPI tetrasaccharide can be seen as a sequence of independent glycosidic linkages. The GPI variants in this study show a similar conformational pattern where the differences in the overall 3D conformation of the GPIs largely arise from differences in rotamer populations at the Man2-*α*(1 *→* 6)-Man1 linkage. In the smaller GPI types, i.e. GPIs 1 and 4, the relative rotamer populations of the Man2–Man1 linkage followed the same order of preference as was seen previously in GPI glycan core in solution and membrane—*gg > gt > tg*. However, in the GPIs with longer branches, i.e. GPIs 2 and 3, the population of *gt* rotamer significantly increased at the expense of *gg*, even to the extent where *gt* exceeds *gg* for GPI 3 in POPC, see Table 1. Out of all the free GPIs considered in this study, GPI 3 is the most flexible due to the presence of an additional flexible (1 *→* 6) linkage in its side branch. The case of GPI 3 also demonstrates that although the conformational preferences of the GPIs largely follow the concept of independent glycosidic linkages (Wehle et al. 2012), there can be important exceptions when side chains are sterically forced to interact with the head group region.

Apart from the internal structure, the presentation of glycolipids on membranes, including their orientation, insertion depth, and exposure to solvent, also influences the way they are recognized or targeted by other biomolecular species. Through a thorough structural investigation of GM3 in a lipid bilayer using NMR and MD simulations, DeMarco and Woods (2009) observed that the receptor protein recognizes the most easily accessible head-group residues of GM3. Similarly, with the help of MD simulations, another study on GM1 provided an explanation for certain carbohydrate-binding proteins having evolved to recognize the most frequently exposed glycolipid epitopes (DeMarco et al. 2010). These studies demonstrated that the terminal glycan residues are the binding epitopes for protein receptors as these are presented on the membrane surface. The exposure, and hence the significance, of terminal residues of GM1 and GM3 follows from the stiff *β*(1 *→* 4) and *β*(1 *→* 3) glycosidic linkages in the core structure, resulting in their erect orientation on the membrane. However, the same does not hold true for GPIs that bear a different set of glycosidic linkages (rich of rather flexible *α* linkages) and furthermore, the unique presence of a positively charged glucosamine in their structure, which altogether leads to a rather different appearance of a reclining, yet flexible GPI glycan. Our results from the free GPI simulations reveal a consistent pattern across all the GPIs in the presentation of the side branches on the membrane surface. The side chain residues—GalNAc in 1, GalNAc and Glc in 2, GlcNAc in 3, and GalNAc and mPEtN in 4—are relatively less membrane-embedded, more hydrated and more solvent-interactive than the other residues, and therefore, could be the most accessible epitopes for recognition. However, the galactose (Gal) residue in 3 makes an exception by interacting profusely with the membrane heads, possibly because of the longer and more flexible (1 *→* 6) linkage in the side branch. The importance of the side branches has also been demonstrated in experimental studies. Using surface plasmon resonance, Debierre-Grockiego and coworkers showed that *T. gondii* GPIs strongly bind to galectin-3, a protein whose expression is a necessary precursor to TNF-*α* production by GPI-stimulated macrophages. Because galectin-3 binds specifically to *β*-galactosides, this finding implies that it associates with the residue GalNAc in *T. gondii* GPI (Debierre-Grockiego et al. 2010). No difference in binding was observed in the presence of an additional Glc residue (*T. gondii* LMW GPI). However, other studies report a striking difference in the immunogenicity of the 2 GPI variants of *T. gondii*. Azzouz et al. (2006) conducted in vitro macrophage activation with *T. gondii* isolates to reveal that the protein-free *T. gondii* LMW GPIs generated 4–5 times more TNF-*α* than the protein-bound *T. gondii* GPIs. This finding was further corroborated by another independent study using synthetic protein-free *T. gondii* GPIs whereupon difference between the 2 GPI variants was observed in the intensity of binding to IgG and IgM antibodies from the sera of infected patients. *T. gondii* LMW GPI bound more strongly to both antibody types than did *T. gondii* GPI, thereby, qualifying as a suitable biomarker to differentiate between latent and acute phases of infection that differ in the levels of IgM antibodies (Gotze et al. 2014). Interestingly, they also showed that a minimal epitope comprising the full side branch—Man1-GalNAc-Glc—bound to the antibodies to a similar extent as the full length GPI. Our results are in agreement with these findings in that the side branches of GPI 2, GalNAc-Glc, are the most solvent-accessible residues, and thereby, are potential antigenic epitopes. As we observed from our simulation data that the side branch of GPI 3 is less solvent-exposed than that of GPI 2, due to the more flexible glycosidic linkage, we hereby predict that GPI 2 should be far more immunogenic than GPI 3 in the same setting.

In humans, an enzyme called GPI transamidase (GPIT) catalyzes the attachment of the GPI anchor to proteins. Vainauskas and Menon showed that mPEtN is required for the recognition of GPI by human GPIT, whereas tPEtN does not play a crucial role (Vainauskas and Menon 2006). In fact, the minimal GPI epitope for recognition by GPIT comprised Ino-GlcN-Man1-mPEtN, although binding was slightly enhanced using the full GPI anchor. These observations align well with our findings showing that mPEtN is more solvent-accessible than tPEtN, irrespective of the host lipid bilayer. Additionally, upon scrutinizing the glycosidic torsion populations of GPI 4, we noted a difference from that of 1 at the Man2-Man1 linkage, as the dominant cluster conformation carried ψ *≈* 90°, as opposed to ψ *≈* 170° in GPI 1. GPIs 1 and 4 differ only through the presence of mPEtN. This difference suggests that conformation of human GPI differs, albeit slightly, from that of *T. gondii* GPI, regardless of the large similarity in their chemical built. There is experimental proof for the absence of cross-reactivity between parasitic and human GPIs, i.e. antibodies raised against parasitic GPIs do not bind with human GPI (Gotze et al. 2014), thereby ruling out the possibility of auto-immune responses. Interestingly, the side chain decorations of GPI glycans are most often found directly bonded to the middle mannose Man1 (especially heavy branches), except when the additional residues are mannoses in which case they branch out from the terminal mannose Man3 (Ferguson et al. 2017). As is evident from our findings that all the GPIs consistently flop down on the bilayer, we deduce that the flop-down conformation facilitates the presentation of the middle portion of the GPI, i.e. Man1 along with its side branches, to the solvent. Our results also provide an explanation for why immune responses to different GPI variants are specific in nature (Tsai et al. 2012). Given that GPI variants differ from each other in their side chain composition, our results imply that specific immune responses are directed against these differing residues that are the most solvent-accessible. Besides, all the side branches were seen to be more embedded in the lipid heads in POPC than in DMPC, implying that in an experimental setting the lipid bilayer type could be varied to test the immunogenicity of the GPIs.

As GPI anchors are often found attached to proteins, it was important to investigate the interactions between GPI and an attached protein. We chose the GFP as the substitute for natural GPI-anchored proteins. GFP by itself does not favorably interact with zwitterionic membranes (such as DMPC and POPC) as is known experimentally (Kim et al. 2013) and also through our control simulations of GFP without the GPI on membrane (data not shown). However, upon attachment to a GPI anchor, GFP is forced to be in closer proximity to the membrane in case of GPI 0, 1 and 3, but not much in case of 2 where considerably greater orientational fluctuation was observed. Irrespective of the GPI type, all GPIs interact with GFP, consequently affecting its orientation. The highly flexible PEtN linker that bridges the GFP to GPI has much to contribute to the oscillating tilt values. The GFP-vs-GPI contact maps reveal that contacts are made mostly at the flexible and disordered loops at the base of the rigid barrel of GFP, and so these interactions would be weak, see Figure 8, thus, contributing to the unstable orientation of the protein. Summing up, although we cannot (for good reasons) rigorously prove the statistical convergence of the GPI-anchored GFPs considered in this work, our data when combined with earlier results, suggest that the GFP can assume a broad variety of orientations, and is still able to interact with the GPI glycan backbone and side chains. Altogether this provides a solid working hypothesis for eventual experimental investigations. Cluster conformations show that irrespective of the orientation of GFP, GPIs lie close to and along the plane of the membrane, consequently pulling the protein close to the membrane. We observed that orientation of the attached protein may differ largely even with seemingly minute differences in the chemical composition of GPIs, irrespective of the same molecular size/weight and level of branching, for example, between GPIs 2 and 3). Hence, using GPI analogs to study the behavior of true GPIs should be avoided.

There are conflicting opinions in literature on whether GPI causes any conformational change in the attached protein. For example, it has been reported that GPI anchor brings about conformational change in Thy1 (Barboni et al. 1995), but not in PrP protein (Zuegg and Gready 2000). As GFP has quite a rigid structure we did not observe any significant conformational changes. Our model of GPI-anchored GFP in a bilayer appears similar to the structure of GPI-anchored VSG of Trypanosomes in a membrane deduced by Homans et al. where the GPI anchor, by spanning across the membrane surface, acts as a space filler between the protein and the membrane (Homans et al. 1989). Another study on the GPI-anchored VSG reported that the galactose side branch increases the volume of the C-terminal domain by associating closely with the protein (Jones et al. 2008). These findings coupled with our GPI 3 results suggest that the membrane-protective property of the VSG is enabled by close contacts between GPI and both the lipids and the protein, whereby the reclining protein covers the membrane and shields it from other biomolecules (Fig. 9). Our results also bring to light an interesting dual characteristic of the GPI side branches when attached to GFP, where on the one hand they are in close proximity to the membrane, and on the other are the most solvent-accessible residues. This suggests that despite protein-attachment, the side chain residues are still exposed, and could be potential chemical targets for drug therapies.

## Conclusions

Using atomistic MD we have investigated and compared the conformational behavior of parasitic and mammalian protein-free and protein-attached GPIs inserted into pure bilayers of DMPC and POPC by extending our previously reported model of the GPI anchor core. Our results from the simulations of free GPIs indicate that the side-chain residues that branch out from the middle mannose Man1, whether sugar residue or phosphoethanolamine, are the most solvent-accessible epitopes of the whole GPI, and thus, are potential targets for recognition of parasitic GPIs by immune cells or of human GPI by the GPI transamidase. In this way, we are able to provide a new insight into the importance of the side-chain residues that has been demonstrated in experiments. The orientation and presentation of the GPI anchor with respect to lipid bilayers is qualitatively consistent across all GPI variants in that the GPIs lie parallel to the bilayer and are in contact with the lipid head-groups. Such a flop-down orientation allows for the side chain residues to project out into the solvent making them readily accessible for molecular recognition. The flop-down conformation has further consequences in that the lipid composition also makes a marked difference in the recognition process, as it was observed that GPIs are more buried in the lipid headgroup region of POPC than DMPC.

We conclude from the GPI-anchored GFP simulations that the orientation of the protein on the membrane depends on several factors: (i) the molecular weight of GPI, (ii) the type of glycosidic linkage between residues, (iii) the region of the protein in contact with GPI (whether a rigid or flexible patch), (iv) the flexibility of the PEtN linker, and (v) the lipid composition of the bilayer. The use of GFP as the attached protein helped to exclude direct protein-membrane interactions, and instead reflected how the conformational preferences of the GPI glycan and its interactions with the GFP influence the latter’s lateral orientation. This enables us to demonstrate that the presentation of the protein depends even on small differences in composition between the GPI variants. We believe that this numerical atomistic model to describe GPIs and GPI-anchored proteins in a modular way is a useful tool for further investigation of these complex systems.

## Computational methods

### MD simulations

All the MD simulations were conducted using the simulation package GROMACS version-2018.3 (Abraham et al. 2015). Lipid bilayers, including the lipid tail of the GPI anchors, were modeled with Lipid14 (Dickson et al. 2014) parameters. The glycan heads of all the GPI anchors in this study were designed with GLYCAM06 (Kirschner et al. 2008), an AMBER-compatible force-field designed exclusively for carbohydrates. The glycan head and the lipid tail were linked together via a hybrid inositol–phosphoglycerol moiety that was constructed in our previous work (Banerjee et al. 2018). The protein GFP was constructed with AMBER’s latest force-field for proteins called AMBERff14SB (Maier et al. 2015). These 3 force-fields belong to the AMBER family and are known to be compatible with each other. Explicit waters modeled with TIP3P (Jorgensen et al. 1983) were used to represent the aqueous phase. Simulations of bilayers were set up in rectangular boxes of dimensions (6.5 × 6.5 × 17) nm^3^ for the free GPI systems and (12.5 × 12.5 × 22) nm^3^ for the GPI-anchored GFP systems. The smaller protein-free GPI systems consisted of 1 GPI anchor per leaflet in pure bilayers of (8 × 8) lipids, whereas the bigger systems had 1 GPI-anchored GFP embedded in 1 leaflet of pure (16 × 16) lipid bilayers. The pure bilayers considered in this work were of DMPC and POPC. Charged systems were neutralized by adding Na^+^ or Cl*^−^* counter ions. System construction was achieved with the LEap facility of AMBER, following which the topology and structure files were converted to GROMACS’ format using a modified version (Wehle et al. 2012) of the script originally written by Sorin and Pande (Sorin and Pande 2005). For every protein-free GPI system, 3 independent 1 μs long simulations were performed with 1 GPI in each leaflet of the bilayer, thus resulting in 6 μs worth of conformational sampling for the GPIs. Similarly, 4 independent 1 μs long trajectories amounting to 4 μs were carried out for the GFP–GPI systems. Table 2 details the summary of all the simulations set up in this work.

**Table 2:** . Summary of the simulations performed in this work.

GPI	Lipid bilayer	#GPIs in bilayer	Time (μs) #runs × #GPIs × time	Total time (μs)
1	DMPC	2	3 × 2 × 1	6
	POPC	2	3 × 2 × 1	6
2	DMPC	2	3 × 2 × 1	6
	POPC	2	3 × 2 × 1	6
3	DMPC	2	3 × 2 × 1	6
	POPC	2	3 × 2 × 1	6
4	DMPC	2	3 × 2 × 1	6
	POPC	2	3 × 2 × 1	6
GFP–GPI				
0	DMPC	1	4 × 1 × 1	4
	POPC	1	4 × 1 × 1	4
1	DMPC	1	4 × 1 × 1	4
	POPC	1	4 × 1 × 1	4
2	DMPC	1	4 × 1 × 1	4
	POPC	1	4 × 1 × 1	4
3	DMPC	1	4 × 1 × 1	4
	POPC	1	4 × 1 × 1	4

To begin with, each system was subjected to energy-minimization through 10,000 steps of the conjugate-gradient method with steepest descent invoked every 1,000 steps. Next, NPT equilibration was performed for 100 ps at temperature 100 K while restraining the positions of the solute molecules so as to relax the water molecules around the solute. The temperature was then ramped up to 303 K for another 100 ps while still restraining the solute. After equilibration, the restraint was released to carry out production run. Temperature was maintained at 303 K by a Langevin thermostat with a coupling constant of 1 ps. Semi-isotropic pressure coupling was applied with a time constant of 1 ps by the Berendsen Barostat (Berendsen et al. 1984) to maintain the pressure at 1 bar. The linear constraint solver algorithm (Hess et al. 1997) was employed to constrain all the bonds containing hydrogen. The leap-frog stochastic dynamics integrator (Van Gunsteren and Berendsen 1988) was used at a time-step of 2 fs to solve the equations of motion to propagate the system. The Particle-Mesh Ewald (PME) algorithm was used to describe electrostatic interactions (Tom et al. 1993). The cut-off for both Coulombic and van der Waals potentials was 1 nm.

### Charge derivation for phosphoethanolamine

To ensure force-field compatibility with the attached GPI glycan, we followed the approach of the original GLYCAM paper (Kirschner et al. 2008) to derive charges for the phosphoethanolamine (PEtN) residue that is attached to the terminal mannose Man3 of GPI. PEtN along with Man3, shown in Figure S11 was considered for the calculation of atomic partial charges for the PEtN residue. Geometry optimization was conducted at a high level of theory—B3LYP/6-31++g(2d,2p)—including diffuse orbitals to account for the charges. The main chain bonds of the molecule were maintained in an all-trans arrangement throughout the calculation. This was to avoid transfer of positively charged H^+^ from NH^+^ to negatively charged O*^−^* of PO*^−^*_4_ . Partial charges were then derived by applying the restrained electrostatic potential (RESP) method for charge fitting. The electrostatic potential was obtained from the optimized geometry at the HF/6-31*G level of theory. As the designed system is meant for condensed-phase simulations, a restraint weight of 0.01 was applied for the RESP charge fitting. Charges on alkyl hydrogens were fixed at 0 to maintain consistency with GLYCAM parameters. GLYCAM’s charge derivation protocol offers modular blocks of sugar residues for building long glycans with variable branching. As a result, every terminal sugar residue carries a net charge of 0.194. The total charge on the PEtN linker was also 0.194, consistent with GLYCAM’s formula. The atom types and bonded parameters (bonds, angles, and dihedrals) assigned to the linker were taken from GLYCAM’s database. In our previous work, partial charges were derived similarly for the PEtN linker that bonds with GPI at one end and to protein on the other. The remaining details of the methodology can be found in ref (Banerjee et al. 2018). The atomic charges thus derived are listed in Table SI of the SI.

### Cluster size calculation

Cluster analysis on the conformation of GPIs was performed using the method described in the work of Daura et al. (1999). The analysis was carried out on a concatenated trajectory of all independent simulations of each GPI variant under study. For each protein-free GPI system, 5,000 structures were used for the calculation. 2,500 structures from each protein-attached GPI system were subjected to clustering. In this method, the root-mean-square deviation (RMSD) of atom positions between every pair of structures is calculated. The number of neighbors, or members, of a cluster is counted based on RMSD value within a given cut-off. The structure with maximum neighbors is picked as the center of that cluster. This process of filtering is done on repeat until there are no structures left in the pool. We applied a cut-off of 0.3 nm, which is higher than the usual cut-off 0.1 nm used for proteins. Carbohydrates are a lot more flexible than proteins, and a tight cut-off value would not give meaningful results. The GROMACS utility *gmx cluster* was employed for this calculation.

## Abbreviations

GPI, glycosylphosphatidylinositol; GPI-AP, GPI-anchored protein; GFP, green fluorescent protein; PEtN, phosphoethanolamine; tPEtN, terminal phosphoethanolamine; mPEtN, middle phosphoethanolamine; PGL, phosphoglycerol; GPIT, GPI transamidase; *T. gondii*, *Toxoplasma gondii*; *T. gondii* LMW, *Toxoplasma gondii* low molecular weight; *T. congolense*, *Trypanosoma congolense*; VSG, variant surface glycoprotein; MD, molecular dynamics; RESP, restrained electrostatic potential.
